# Dentistry, Its History, Present Status Claims and Relations

**Published:** 1873-09

**Authors:** Wm. F. Thompson


### DENTAL REGISTER.

VOL. XXVII.] SEPTEMBER, 1873. [No. 9.

### DENTISTRY, ITS HISTORY, PRESENT STATUS CLAIMS AND RELATIONS.

*Read before the Oregon State Dental Society, June 25th, 1873, by Dr. Wm. F. Thompson*

*Mr. President and Gentlemen:*

The subject of my thesis, is one that involves so much of interest to our profession, that I am at a loss in what shape to present it. The old adage, that "He who once puts hand to the plow should never look back," as a general rule is good, but we must make this the exception, for it is but proper, that we should trace back to its very fountain head, a vocation that is not only our sustenance, but *should be* our *professional pride.* To investigate from whence we sprang, our advancement, present standing, and prospective future, is a duty we owe to ourselves, and those dependent upon us. Ours is a profession that will always honorably represent its practitioners, so long as it is not misrepresented by them, or debased for purely selfish motives. I cannot but pronounce it one of the lost arts. That it is a newly created thing, to satisfy the demands of newly discovered disorders, is most improbable ; for if we may believe facts given in history, so long ago as four hundred years before the Christian era, the art of dentistry appears to have been practiced in Egypt. In the ancient tombs of this people, artificial teeth were found, also the teeth of mummies filled with gold. During the eighteenth century, the attention of medical men in France and England was directed to the subject, and a number of works were published devoted exclusively to the art. The treatise of John Hunter, 1771-8, laid the foundation of the English school of dentistry. The subject, however, was treated philosophically, but not practically, for none of these were practical dentists. Other publications of a later date, served rather to investigate the nature of the diseases to which the teeth are subject, than the best method of treating them.

Medicine and dentistry are so closely allied, that the students of each, have in many cases to study the same books, and draw their information from the same source. Like parallel lines frequently blending, stand the two professions today. In the inter-change of ideas, both are oft-times benefited, and never in any case, by such contact are they injured. The dentist should as thoroughly understand his patients, their habits, etc., as the physician. They each stand in a position to *demand* that *perfect confidence*, which *only* can insure complete success. This accomplished, and one half the desired results have been attained; but with that confidence withheld, no operator knowing it, should bestow his services upon so unworthy and distrustful an object. Dentistry ceases to be mechanical, and becomes thoroughly scientific and professional, when treatment of the teeth demands a comprehension of the relations which the various organs sustain to the general system, and their mutual dependence upon each other. Consequently he who practices the art should pause before operating, to consider conscientiously the proper course to pursue. So closely connected are the nerves of the teeth with all other parts, and so perfect in their adjustment, that like the telegraph system so admirably adapted to our wants, the nerves are so many electric wires, distributed throughout the body, and when any part is touched by disease, the whole physical system is made to thrill with pains, to the confusion sometimes of those who are applied to for relief. A knowledge of medicine, and the diseases these organs are most subject to, is positively necessary to the scientific man, and thoroughly practical dentist. His knowledge of anatomy should not be confined to the teeth only, any more than a student in geography should confine his geographical information to one state; for every part is so essentially connected with the whole, that to comprehend and diagnose a local disease, he must thoroughly understand the influences that may be brought to bear from an outside source. Our science involves an acquaintance with the anatomical relations of the organs of the mouth, with all parts of the system; and the position of the nerves which communicate their impressions to the brain; for we must admit that many of the ills which "flesh is heir to," are purely mental. The teeth may be affected by remote causes, the remedy for which is to be applied to other organs. Throughout the whole system, like a net-work, are the nerves entwined, and are affected frequently in the teeth, by the diseases of distant members; and an operator unskilled might diagnose the case entirely wrong, and instead of alleviating, might aggravate, causing his victim of misplaced confidence most exquisite suffering—troubles which are produced by carelessness, or indifference concerning that which is of mutual interest to both. No conscientious dentist will permit those seeking his services, to turn him one hair's breadth from the position he may deem right; for what at the time would seem a pecuniary gain, might prove a lasting curse, extending through his whole practice. To permit his patient to dictate the manner in which the work should be done, and what fee is equitable, must necessarily involve both patient and operator in trouble.

Right here I wish to say something concerning that much written about, and talked of subject: "Fees," and *things pertaining thereto.* Commercially speaking, every marketable article, in price, is regulated by the unyielding, and generally accepted law of supply and demand. To a certain extent this is true, viewed in a professional light; what the masses every where ask, is "value received," and when they have once learned *when* that *want* can be supplied, they as a rule unquestioningly accept it. That man, who courteous, yet *self-reliant*, and with the *faith big in him*, insists that justice be done, not only his patients, but himself, must and will ultimately reap the reward so justly his due. The question of fees should be a secondary consideration, but alas! how many there are who make it the first. Services rendered should always regulate the fee, and never in any case should an operator give abortive work, because his patient fails to comprehend its worth. Better let that patient pass into other hands, for I speak advisedly when I say that that class is always unremunerative, and what proves more discouraging to an earnest worker is, that they are with hardly an exception, unappreciative people. Desiring to get much for little, they are always expecting to get "beat" and generally are not disappointed; but should they get full value, are surprised and still suspicious, because as a rule they are victims to their own greed, and fall into the hands of unprincipled operators, who use them for a purpose. Consequently that both parties get value received, a fair equivalent should be demanded; and when the dentist has put forth his best efforts to produce the desired results, let him not disgrace his profession, or forego his just rights by accepting a laborer's wages. As I have said, competition should not be in price, but in work; this is honorable, and will always bring satisfactory results to those practicing it; an appreciative public will generally remunerate where there is true merit, and severely criticise where there is not. It is essential above all things that an operator be courteous and gentlemanly; for so close is the relationship between those he serves and himself, that any little discrepancy of character, would be noticed and commented upon to his disadvantage. He may advertise, but his advertising should be done in the mouth; here only will it permanently recommend him to favor.

As I close this paper, permit me to add one word concerning professional courtesy. Its influence upon the individual practitioner, is to elevate him to a higher sphere of usefulness. It harmonizes all his acts to his own well being ; while it does no injustice to those competing with him. This is as it should be, and will, if each comprehend the other's rights and guard against infringement. Professional jealousies have been too much the stumbling-blocks to our own advancement. Unity of action will always strengthen, while a divided sentiment—whether expressed or not—must necessarily weaken the chances of success in any work engaged. For young practitioners who feel how slow the world is to appreciate, I have the greatest sympathy. The trials connected with my early experiences, are among the most vivid recollections of the past. My first effort was made in Cincinnati, 1860, and for six months my practice did not remunerate me the outlay o office rent. As I was at that time quite young, it might have been attributed to youth ; to some extent this was probably true, but not wholly, for there were radical changes needed, that I did not at that time comprehend. It amused me, while it mortified my youthful vanity exceedingly, to have parties call for the dentist, and when I assured them that I was that individual, have them repeat the question, thinking I did not understand. I estimated my little learning at that time, much higher than I do my present understanding of things. I felt deeply the injustice—as I then thought—an unfeeling public meted out to me ; of course I was in error, and many things that then seemed wrong, experience has taught me were right. This experience comes to all, sooner or later, and although dearly bought, and bitter, frequently proves a great benefit; for it is here that theory without practice, is shown to be unavailing, and pretension without merit, a flimsy screen that must come down, to the confusion of those assuming it. The longer one practices, the less confident does he become of his superior merit over others, and more doubtful that he is square up to the mark, as he should be. And if he discovers—as he thinks,—some bright idea, he is more apt than otherwise, to find some one has occupied the ground before him. Our vocation being a sedentary one, "blue Mondays" will come occasionally, and at such times, we are apt to think the world is not just what it should be. Whether it is or not, I question ; this however must be decided by others. but it is self evident then, that some sovereign cure is needed. It is without doubt the most trying of any of the professions, more soul and body trying, eating out one's very vitals, turning the current of his life-blood backward, making him prematurely old. This is not in the least an overdrawn picture, and with the facts staring us in the face, is it not better to work together in harmony, each extending to the other the helping hand of true sympathy, that results may be attained not only for our own good, but that our profession may continue to advance with the same rapid strides, it has up to the present time ?

In educating ourselves to the standard we should occupy we are raised to a pure atmosphere, made more generous, and are less likely to criticise the short-comings of others, because the better able to understand our own defects. In conclusion, let us throw the heart's mantle of charity over the faults of others, that they alike may be charitably disposed towards us.

